# Analysis of the current status and influencing factors of health literacy regarding unintentional injuries in young children among parents in ethnic regions of Western China

**DOI:** 10.3389/fpubh.2026.1752273

**Published:** 2026-03-24

**Authors:** Yuqi Shen, Zhi Zeng, Qingping Ma, Xuemei Deng, Dan Wen, Li Wan

**Affiliations:** 1 Intensive Care Unit, Mianyang Central Hospital, School of Medicine, University of Electronic Science and Technology of China, Mianyang, Sichuan, China; 2 Beichuan Qiang Autonomous County Education Development Institution, Mianyang, Sichuan, China; 3 Mianyang Children's Palace Kindergarten, Mianyang, Sichuan, China; 4 Department of Nursing, Mianyang Central Hospital, School of Medicine, University of Electronic Science and Technology of China, Mianyang, Sichuan, China

**Keywords:** children, current status, health literacy, influencing factors, unintentional injuries

## Abstract

**Objective:**

To comprehend the present state of health literacy concerning unintentional injuries among parents in ethnic regions of Western China and to analyze the associated influencing factors, offering a reference for the formulation of strategies aimed at enhancing health literacy for unintentional injuries among parents of young children and for refining the management of safety education for young children.

**Methods:**

Cluster sampling was employed to select parents from 18 kindergartens in a specific ethnic region of Western China as the participants of the study. A general information questionnaire and a self-mad health literacy assessment scale for unintentional injuries in young children were utilized for the status survey. Data were statistically described and analyzed using SPSS 27.0 software. Univariate analyses and multivariate binary logistic regression were performed to identify factors associated with adequate health literacy.

**Results:**

54.79% of the parents possess health literacy regarding unintentional injuries in young children. Univariate analysis revealed statistically significant differences (*p* < 0.05) in the child’s grade, parent’s gender, educational level, number of children, residential area, occupation, living area size, monthly family income, experience with relevant knowledge training, self-learning experience, and willingness to receive training. Binary Logistic regression analysis also indicated statistically significant differences (*p* < 0.05) in the parent’s gender, residential area, occupation, number of children, experience with training on relevant knowledge, and self-learning experience.

**Conclusion:**

Parental health literacy regarding unintentional injuries in young children in ethnic regions of Western China is generally low, particularly in rural areas and among unemployed parents. Based on this, safety education and training programs for parents can be developed to bolster their awareness of risk prevention and control, encourage self-directed learning behaviors, and enhance their health literacy in this area.

## 1 Introduction

**Figure 1:**
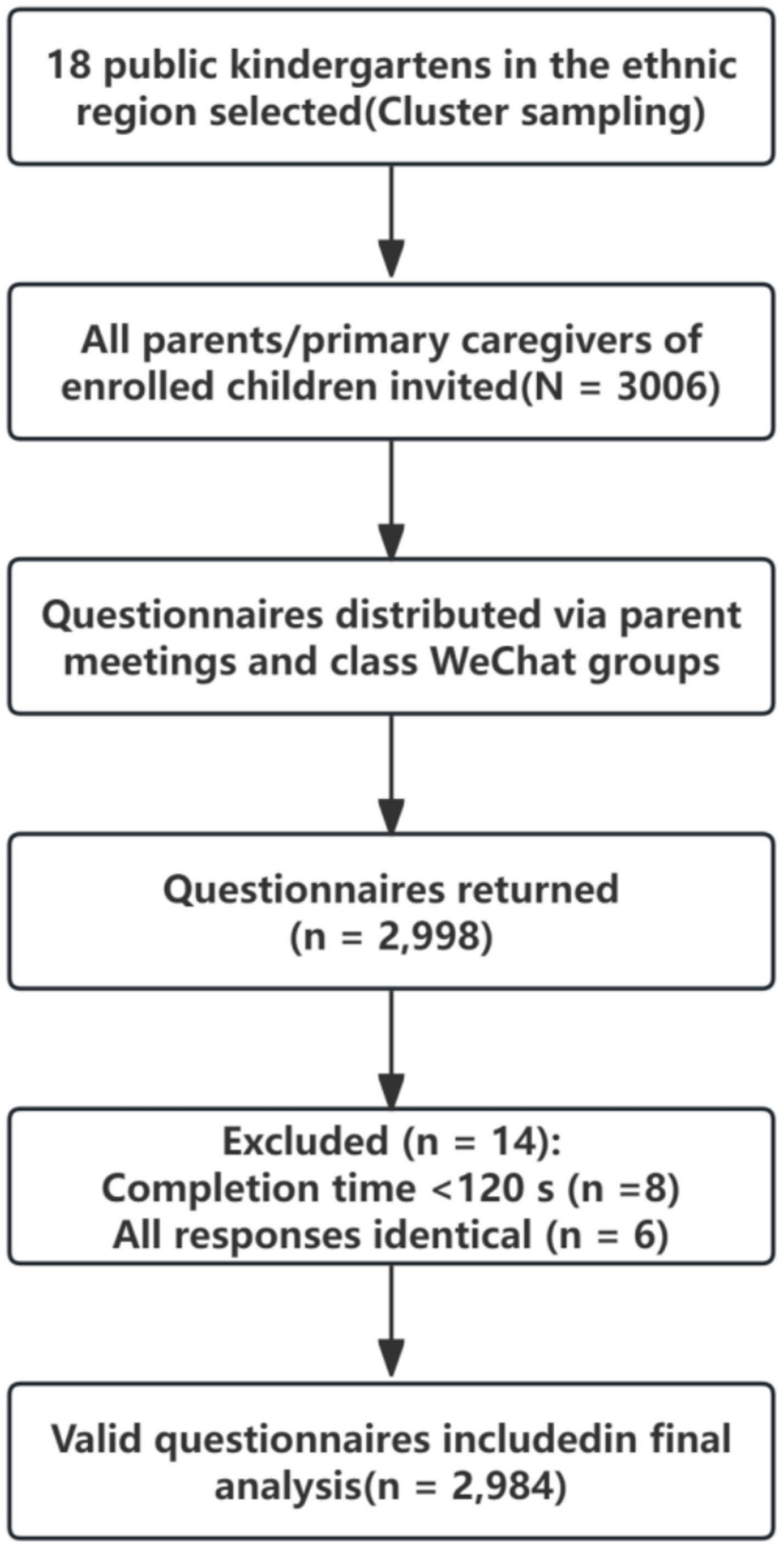
Flowchart of the sampling procedure and participant selection process.

Unintentional injuries are the leading cause of death among Chinese children, claiming over 50,000 lives annually and accounting for 40–50% of all-cause child mortality, with the highest proportion of such deaths occurring in the 1–4 age group at approximately 33% (1, 2). It is worth noting that the locations where child unintentional injuries occur are mainly concentrated at home (65.70%), and 87.45% of these accidents happen with parental supervision (3). The next most common locations are public places and roads/streets (24%), while schools account for only 5.08% (4). The challenge of limited parental health literacy in preventing child unintentional injuries is a global concern, not one unique to China. In the United States, for example, preventable injuries remain the leading cause of death for children. In 2022, over 9,000 families lost a child to such an event, and nearly 5.6 million children are treated in emergency departments for often serious, life-altering injuries each year (1). These stark figures underscore a universal vulnerability. Therefore, understanding the specific determinants of parental health literacy within the unique socio-cultural context of Western China’s ethnic regions can offer valuable insights that extend beyond national borders, informing intervention strategies for similar settings globally. This phenomenon reflects the critical role of parents in child care, as well as the deficiencies in parents’ safety awareness and care abilities. Parental health literacy refers to their ability to obtain, understand, and use health information to maintain and promote the health of themselves and their family members (5). Studies have shown that parental health literacy levels are closely related to their care abilities, with parents who have higher health literacy being more effective in preventing and managing child unintentional injuries (6). The “China Children’s Development Program (2021–2030)” emphasizes the need to reinforce the concept that parents or other guardians are the primary persons responsible for children’s health. The level of parental health literacy not only directly affects the physical and mental health of their children but also significantly influences the children’s own health literacy levels (7, 8). Therefore, improving parental health literacy is an important way to reduce child unintentional injuries (Figure 1).

The 2022 China Education Statistics Yearbook reveals a significant disparity in educational levels between the western ethnic regions and the eastern regions of China (9). Parents’ awareness and prevention capabilities regarding unintentional injuries to children are generally low, which is one of the primary reasons why the all-cause mortality and unintentional injury mortality rates among children in rural and western areas of China are markedly higher than those in urban and eastern areas (10, 11). Data indicates that from 2016 to 2022, the all-cause mortality rates for children under 5 in rural areas and western regions were 7.3 and 8.4%, respectively, while those for urban children, central regions, and eastern regions were only 4.0, 4.1, and 1.1%; among these, in 2022, injuries were the leading cause of death for children under 5, accounting for 23.1% of all-cause mortality. From 2016 to 2022, the incidence rate of unintentional injuries as the cause of death for children under 5 was 1.2%, with the eastern region at 7.6%, the central region at 1.1%, and the western region having the highest proportion of deaths due to unintentional injuries at 3.5% (12). However, existing research has primarily focused on the health literacy of specific groups such as the older population, women, and adolescents, with relatively scant research on the health literacy of parents of young children, particularly in ethnic regions. Although some studies have examined the epidemiological characteristics of unintentional injuries to children, there is still a lack of in-depth exploration of the relationship between parental health literacy and unintentional injuries to children (12). Therefore, this study aims to understand the health literacy of parents in ethnic regions of Western China regarding unintentional injuries to young children and to analyze its influencing factors, in order to provide a scientific basis for targeted safety education and management of young children in ethnic regions.

## 2 Method

### 2.1 Study design and participants

This community-based cross-sectional study was conducted in a specific ethnic region (predominantly Qiang ethnicity) of Western China. Using a cluster sampling method, all primary caregivers (“parents”) of children from 18 public kindergartens were invited to participate in September 2024.

Inclusion criteria: (1) Primary caregiver of a child aged 3–6 years; and (2) Ability to comprehend and complete the Chinese questionnaire independently.

Exclusion criteria: (1) Completion time <120 s; and (2) all responses identical.

### 2.2 Sample size calculation

The sample size was calculated using the formula for estimating a population proportion: *n* = Z^2^ × *P*(1 − *P*)/d^2^. Based on a pilot study (*n* = 50) where 48% of parents had adequate health literacy (*p* = 0.48), with a 95% confidence level (Z = 1.96) and a margin of error (d) of 0.03, the initial calculated sample size was *n* = (1.96)^2^ × 0.48 × 0.52/(0.03)^2^ ≈ 1,065. To account for the cluster sampling design, a design effect of 1.5 was applied, yielding an adjusted sample size of 1,065 × 1.5 ≈ 1,598. Inflating this by an anticipated 15% non-response/invalid rate resulted in a final minimum required sample size of 1,598 × 1.15 ≈ 1837. Our final sample of 2,984 valid responses exceeded this requirement. The design effect of 1.5 was estimated based on the assumed intra-cluster correlation coefficient (ICC) from similar studies on parental health behaviors (13) and the average cluster size in our pilot study.

This study has been approved by the Ethics Committee of Mianyang Central Hospital (Approval No.: S20240227-01), and has obtained the informed consent and organizational support of the education administrative department of the ethnic region.

### 2.3 Measures and tools

Data were collected via an online questionnaire on the Wenjuanxing platform.

#### 2.3.1 Socio-demographic questionnaire

The research group developed the general information questionnaire through literature review, encompassing two dimensions: (1) Basic information of children: gender, age, grade; and (2) Basic information of parents: gender, age, education level, number of children, marital status, residential area, occupation, living area, and monthly family income, among others.

#### 2.3.2 Self-developed health literacy assessment scale for unintentional injuries in children

The research team, referring to the “Parental Safety Literacy Assessment Tool Framework for the Prevention of Childhood unintentional Injuries” constructed by Kaiyue Chen et al. (14), developed a health literacy assessment scale for unintentional injuries in young children. The scale comprises three dimensions: knowledge, attitude, and behavior, with a total of 59 items and a maximum score of 195 points. (1) The knowledge survey section consists of 25 items, all of which are multiple-choice questions. One point is awarded for a correct answer, and zero points for a wrong answer or not knowing, with a total score of 25 points; (2) The attitude section consists of 13 items, using the Likert 5-point scoring method, ranging from “strongly disagree” to “strongly agree,” scored from 1 to 5 points, all positively scored, with a total score of 65 points; and (3) The behavior section consists of 21 items, also using the Likert 5-point scoring method, ranging from “strongly disagree” to “strongly agree,” scored from 1 to 5 points, all positively scored, with a total score of 105 points. The scale refers to the unified scoring standard for health literacy monitoring (15), with a score of 80% of the total score, i.e., 156 points, as the threshold. A total score greater than 156 points is defined as possessing health literacy for unintentional injuries in young children, meaning that parents have a good grasp of relevant knowledge, a positive attitude, and the ability to prevent unintentional injuries in young children. The Cronbach’s *α* coefficient of the self-made health literacy assessment scale for unintentional injuries in young children is 0.801, and the Cronbach’s *α* coefficients for the dimensions of knowledge, attitude, and behavior were as follows: 0.767, 0.852, 0.872, indicating good internal consistency. Content validity was established by a panel of experts (two pediatricians, two public health scholars, and two early childhood educator), who reviewed the items for relevance and comprehensiveness. Construct validity was assessed through confirmatory factor analysis. The full assessment scale is provided as Supplementary File 1.

### 2.4 Data collection and quality control

This study gathered data using the Wenjuanxing software. With the organizational backing of the education administrative department in a specific ethnic region of Western China, the research team carried out offline centralized training for the relevant staff of 18 kindergartens. They explained the objectives and significance of the study, as well as the survey process. The kindergarten staff guided parents in completing the questionnaires during school-wide parent meetings, and the teachers distributed electronic questionnaire QR codes in the class parent groups. Logical checks were incorporated into the questionnaire design (including numerical value ranges, allowed values, answer frequency limits, unanswered reminders, etc.) to minimize the creation of invalid questionnaires. To avoid multiple submissions by the same parent, the questionnaire was configured to permit only one submission per Internet Protocol (IP) address, and all fields were set as mandatory. Refer to Table 1 for further details.

**Table 1:** Univariate analysis of factors included in the final multivariate model (*n* = 2,984).

Variable	Total *n* (%)	Adequate HL *n* (%)	Inadequate HL *n* (%)	χ^2^	*p*-value
Parent’s gender				10.537	0.001
Male	584 (19.57)	291 (49.83)	293 (50.17)		
Female	2,400 (80.43)	1,357 (56.54)	1,043 (43.46)		
Residential area				32.217	<0.001
Urban	2,374 (79.56)	1,356 (57.12)	1,018 (42.88)		
Rural	610 (20.44)	279 (45.74)	331 (54.26)		
Parent’s occupation				78.871	<0.001
Farmer	183 (6.13)	71 (38.80)	112 (61.20)		
Government official	106 (3.55)	70 (66.04)	36 (33.96)		
Corporate employee	713 (23.93)	440 (61.71)	273 (38.29)		
Public institution staff	441 (14.78)	235 (53.29)	206 (46.71)		
Self-employed/Business owner	426 (14.28)	254 (59.62)	172 (40.38)		
Retired	21 (0.70)	13 (61.90)	8 (38.10)		
Unemployed	213 (7.14)	95 (44.60)	118 (55.40)		
Freelance	575 (19.27)	310 (53.91)	265 (46.09)		
Other	306 (10.25)	152 (49.67)	154 (50.33)		
Number of children				9.484	0.009
1	1,600 (53.62)	843 (52.69)	757 (47.31)		
2	1,318 (44.17)	758 (57.51)	560 (42.49)		
≥3	66 (2.21)	34 (51.52)	32 (48.48)		
Relevant knowledge training experience				124.852	<0.001
Yes	894 (29.96)	614 (68.68)	280 (31.32)		
No	2090 (70.04)	1,029 (49.23)	1,061 (50.77)		
Self-directed learning experience				191.308	<0.001
Yes	1,570 (52.61)	1,028 (65.48)	542 (34.52)		
No	1,414 (47.39)	616 (43.56)	798 (56.44)		

Data processing personnel have all undergone specialized training to ensure their familiarity with the questionnaire completion requirements and data collection processes. During the data export phase, to ensure the accuracy and integrity of the data, a method combining dual independent export and review was adopted, and all data underwent two rounds of checking and verification.

### 2.5 Statistical analysis

Data were analyzed using SPSS 27.0. Descriptive statistics were presented as frequencies (percentages) and mean ± standard deviation. Group differences were examined using Chi-square tests. Variables with *p* < 0.1 in univariate analysis were entered into a multivariate binary logistic regression model (Forward: LR method) to identify independent factors associated with adequate health literacy, reported as Odds Ratios (ORs) with 95% Confidence Intervals (*CI*s). *p* < 0.05 was considered significant.

## 3 Result

### 3.1 Univariate analysis of factors associated with health literacy

This survey compiled a total of 2,998 questionnaires, of which 2,984 were deemed valid, resulting in a questionnaire validity rate of 99%. Among the valid responses, there were 584 males (19.57%) and 2,400 females (80.43%). The predominant age group of caregivers was 31–40 years (68.50%). Overall, 1,635 parents (54.79%) possessed adequate health literacy regarding unintentional injuries in young children, while 1,349 (45.21%) did not. Univariate analysis revealed that several variables were significantly associated with health literacy levels (*p* < 0.05), including the child’s grade, parent’s gender, educational level, number of children, residential area, occupation, living area size, monthly family income, experience with relevant knowledge training, self-directed learning experience, and willingness to receive training. Table 1 presents the univariate results for the six variables that were subsequently identified as independent predictors in the multivariate logistic regression analysis. The complete univariate analysis results for all variables examined are provided in Supplementary Table 1.

### 3.2 Multivariate logistic regression analysis of health literacy in children’s unintentional injuries

Using the presence of health literacy among parents regarding unintentional injuries in young children as the dependent variable, and selecting factors with statistically significant differences from univariate analysis as independent variables, the assignment of multicategorical variables employs dummy variable settings. The analysis indicates that gender, residential area, occupation, number of children, experience with relevant knowledge training, and self-learning experience exhibit statistical significance (*p* < 0.05). Multivariate logistic regression analysis revealed that several factors were independently associated with adequate health literacy. Specifically, female parents were 1.24 times more likely to possess adequate health literacy than males (OR = 1.244, 95% *CI*:1.020–1.517). Parents residing in urban areas had 1.29 times higher odds of adequate health literacy compared to rural residents (OR = 1.292, 95% *CI*:1.009–1.656), which may reflect unmeasured protective factors or community support mechanisms in urban settings. Freelance work parents had significantly lower odds of adequate health literacy (OR = 0.632, 95%*CI*: 0.431–0.925), indicating their vulnerability due to limited access to health information and social resources. Parents with two children were nearly twice as likely to have adequate health literacy compared to those with one child (OR = 1.859, 95%*CI*:1.080–3.200), suggesting that parenting experience may enhance safety knowledge. Furthermore, prior training in relevant knowledge (OR = 1.653,95%*CI*:1.333–2.049) and self-directed learning experience (OR = 2.180, 95%*CI*:1.802–2.638) were the strongest modifiable predictors, highlighting the critical role of educational interventions in improving parental health literacy. Refer to Table 2 for further details.

**Table 2:** Multivariate binary logistic regression analysis of factors associated with adequate health literacy (*n* = 2,984).

Variable	Category	*B*	SE	Wald χ^2^	*P*-value	OR	95% *CI*
Preschool grade level				5.101	0.277		
	Nursery class (ref.)	0	–	–	–	1.000	–
	Junior class	0.234	0.288	0.659	0.417	1.264	0.718–2.224
	Middle class	−0.033	0.257	0.016	0.899	0.968	0.585–1.603
	Senior class	−0.131	0.252	0.269	0.604	0.878	0.536–1.438
	Preschool class	−0.102	0.252	0.164	0.685	0.903	0.551–1.480
Parent’s gender	Male (ref.)	0	–	–	–	1.000	–
	Female	0.218	0.101	4.640	0.031	1.244	1.020–1.517
Parental education level				5.239	0.155		
	Junior high school and below (ref.)	0	–	–	–	1.000	–
	High school/Secondary technical school	−0.10	0.248	0.181	0.671	0.900	0.554–1.463
	University/College	−0.079	0.225	0.123	0.725	0.924	0.595–1.435
	Master’s degree or above	0.143	0.200	0.512	0.474	1.154	0.779–1.708
Area	Rural (ref.)	0	–	–	–	1.000	–
	Urban	0.257	0.126	4.117	0.042	1.292	1.009–1.656
Parent’s occupation				19.853	0.011		
	Farmer (ref.)	0	–	–	–	1.000	–
	Government officials	−0.353	0.223	2.510	0.113	0.703	0.454–1.087
	Corporate employees	0.162	0.251	0.415	0.519	1.176	0.719–1.923
	Staff members of public institutions	−0.107	0.148	0.518	0.472	0.899	0.672–1.202
	Individual business operator	0.253	0.165	2.358	0.125	1.288	0.932–1.781
	Retirement	0.134	0.162	0.678	0.410	1.143	0.832–1.571
	Unemployed	−0.049	0.472	0.011	0.917	0.952	0.378–2.400
	Freelance work	−0.459	0.195	5.572	0.018	0.632	0.431–0.925
	Other	−0.035	0.150	0.056	0.813	0.965	0.719–1.295
Residential floor area				6.301	0.178		
	<61 m^2^ (ref.)	0	–	–	–	1.000	–
	61–80 m^2^	−0.209	0.268	0.607	0.436	0.811	0.480–1.373
	81–100 m^2^	−0.311	0.161	3.722	0.054	0.733	0.534–1.005
	101–120 m^2^	−0.252	0.110	5.297	0.021	0.777	0.627–0.963
	>120 m^2^	−0.171	0.109	2.460	0.117	0.843	0.681–1.044
Monthly household income				4.575	0.334		
	<5,000 Yuan (ref.)	0	–	–	–	1.000	–
	5,000–10,000 Yuan	−0.061	0.165	0.136	0.712	0.941	0.682–1.299
	10,001–15,000 Yuan	−0.172	0.138	1.555	0.212	0.842	0.643–1.103
	15,001–20,000 Yuan	−0.226	0.142	2.522	0.112	0.798	0.112–0.798
	>20,000 Yuan	−0.011	0.160	0.005	0.945	0.989	0.723–1.353
Number of children				6.287	0.043		
	1 (ref.)	0	–	–	–	1.000	–
	2	0.620	0.277	5.012	0.025	1.859	1.080–3.200
	≥3	0.496	0.275	3.252	0.071	1.643	0.958–2.818
Relevant knowledge training	No (ref.)	0	–	–	–	1.000	–
	Yes	0.502	0.110	20.938	<0.0001	1.653	1.333–2.049
Self-directed learning	No (ref.)	0	–	–	–	1.000	–
	Yes	0.779	0.097	64.176	<0.0001	2.180	1.802–2.638
Willingness to receive training	No (ref.)	0	–	–	–	1.000	–
	Yes	−0.122	0.126	0.946	0.331	0.885	0.691–1.132
Constant		−0.706	0.468	2.272	0.132	0.494	–

## 4 Discussion

Preventing unintentional injuries in young children is a crucial aspect of child health care and a central concern for both society and public health (16). This study found that only 54.79% of parents in ethnic regions of Western China possess adequate health literacy regarding unintentional injuries in young children, a figure comparable to the 58.00% rate reported among parents of newborn infants (17). Meanwhile, this study revealed that parents demonstrated relatively high scores in the attitude dimension (62.04 ± 4.38, out of a possible 65), indicating generally positive attitudes toward injury prevention. However, their performance in the knowledge dimension (17.97 ± 3.85, out of 25) and behavior dimension (76.81 ± 12.60, out of 105) was substantially lower. This disparity between positive attitudes and inadequate knowledge/practices suggests that while parents recognize the importance of preventing childhood injuries, they lack the specific knowledge and practical skills necessary to implement effective preventive measures. This gap represents a critical target for intervention and may partially explain the persistently high rates of child unintentional injuries in this region.

### 4.1 Factors associated with parental health literacy

This study identified six factors independently associated with parental health literacy regarding unintentional injuries in young children: parents’ gender, residential area, occupation, number of children, prior training experience, and self-directed learning experience. These findings align with and extend previous research on health literacy determinants.

Gender disparity. Female parents demonstrated significantly higher health literacy levels than their male counterparts (OR = 1.244, 95% *CI*:1.020–1.517). This finding is consistent with studies showing that women typically assume greater responsibility for daily childcare, thereby accumulating more experience and knowledge in managing children’s health and preventing injuries (18, 19). The lower health literacy among fathers highlights a critical gap in shared parenting responsibilities and suggests that safety education efforts have disproportionately reached mothers.

Urban–rural divide. Parents residing in rural areas exhibited lower health literacy compared to urban residents (OR = 1.292 for urban vs. rural, 95% *CI*:1.009–1.656). This disparity likely reflects the scarcity of educational resources and limited health education infrastructure in rural regions (20). Rural parents often have fewer channels for accessing health knowledge, and the scope of available health education is narrower, resulting in weaker capacities for injury prevention and emergency response.

Occupation and socioeconomic vulnerability. Freelance work parents had significantly lower odds of adequate health literacy (OR = 0.632, 95% *CI*:0.431–0.925). This finding may be attributed to their reduced exposure to social resources and health information networks (21). Freelance work individuals may miss opportunities to participate in workplace-based health education and have smaller social networks through which health knowledge typically circulates.

Family structure. Parents with two children were nearly twice as likely to possess adequate health literacy compared to those with only one child (OR = 1.859, 95% *CI*:1.080–3.200). This suggests that parenting experience accumulated over time enhances safety knowledge and practices. Interestingly, while single-child families might be expected to invest more attention in their child’s safety, the experience gained from raising multiple children appears to confer a protective advantage (22).

Modifiable factors: training and self-learning. The strongest predictors of adequate health literacy were prior experience with relevant knowledge training (OR = 1.653, 95% *CI*:1.333–2.049) and self-directed learning (OR = 2.180, 95% *CI*:1.802–2.638). These findings underscore the critical role of educational interventions in improving parental health literacy. Systematic health education can equip parents with essential knowledge and skills for preventing unintentional injuries and responding to emergencies (23). The fact that these are modifiable factors offers clear targets for intervention, particularly given the identified gap between positive attitudes and inadequate knowledge/practices.

The disparities observed in this study—by gender, residence, occupation, and family structure—are consistent with broader patterns of health inequality in China. The China Education Statistics Yearbook reveals significant disparities in child health outcomes between western and rural regions compared to central, eastern, and urban areas, disparities attributed to lower economic development, educational resource shortages, and inadequate parental health literacy (24).

### 4.2 Implications for practice

Our findings point to several actionable strategies for improving parental health literacy, particularly by targeting the modifiable factors identified and addressing the attitude-knowledge-practice gap revealed by the dimensional scores.

Targeting disadvantaged subgroups. The lower health literacy among fathers suggests a need for targeted engagement strategies. Kindergartens could organize “father-child safety workshops” to enhance paternal involvement in injury prevention. For rural parents, who face barriers accessing health information, a combination of digital tools and community-based programs is essential. Mobile health (mHealth) interventions, such as WeChat-based safety education services, can disseminate concise, visual injury prevention content to rural families. These should be complemented by hands-on training programs—lectures, drills, and first aid training—delivered through kindergartens and tailored to rural parents’ needs for basic, practically oriented content (25–28). Given the low knowledge scores observed, such programs should emphasize foundational safety knowledge before advancing to more complex topics. For unemployed parents, community health centers and kindergartens can collaborate to offer free, low-threshold training sessions with flexible scheduling, potentially combined with incentives (e.g., childcare supplies) to encourage participation (29).

Leveraging family experience. Parents with two children demonstrated higher health literacy, likely due to accumulated experience. Peer education models, where experienced parents serve as volunteers or role models, could be piloted to facilitate knowledge sharing among families (30). This approach harnesses existing community resources and creates sustainable support networks. Given the low behavior dimension scores, peer educators could particularly focus on demonstrating and modeling practical safety behaviors in home settings.

Institutionalizing training and self-learning. Given that training and self-learning were the strongest modifiable predictors, regular safety education for parents should be institutionalized. Kindergartens should incorporate injury prevention training into their annual parent education plans, and local education authorities could develop certified online courses to support parents’ self-directed learning (31). The content of such training should address the specific knowledge deficits identified, with particular attention to translating positive attitudes into concrete preventive behaviors. For kindergarten teachers—frontline workers in children’s safety education—systematic training on unintentional injury prevention and emergency response should be provided, with ongoing continuing education and evaluation (32). Teachers can then integrate safety education into children’s daily activities through games and role-playing, helping children master emergency skills and enhance self-protection awareness (33), thereby complementing and reinforcing parents’ efforts.

## 5 Limitations

This study has several limitations. First, the cross-sectional design precludes causal inference; we can only describe associations, not determine directionality. Second, data were self-reported, which may introduce social desirability bias, particularly in the attitude and behavior dimensions. Third, we did not collect data on children’s actual injury history, preventing assessment of the direct link between parental health literacy and injury occurrence. Fourth, the study was confined to a single ethnic region in Western China (predominantly Qiang), limiting generalizability. Fifth, residual confounding from unmeasured variables (e.g., grandparent involvement, healthcare access) cannot be ruled out. Future research should employ prospective cohort or intervention designs with injury incidence as the primary outcome to establish causality and evaluate targeted interventions.

## 6 Conclusion

This study revealed that only 54.79% of parents in ethnic regions of Western China possess adequate health literacy regarding unintentional injuries in young children, with significantly lower levels observed among rural residents, unemployed individuals, and fathers. Parental gender, residential area, occupation, number of children, prior training, and self-learning experience were identified as independent influencing factors. These findings highlight the urgent need for targeted, multi-level interventions that address the specific disadvantages faced by these subgroups. Future efforts should prioritize accessible training programs, promote self-directed learning, and strengthen collaboration among families, kindergartens, and community health services to reduce unintentional injuries in young children.

## Data Availability

The original contributions presented in the study are included in the article/Supplementary material, further inquiries can be directed to the corresponding author.
