# Supplementary material for: Analysis of the current status and influencing factors of health literacy regarding unintentional injuries in young children among parents in ethnic regions of Western China
Source: Front Public Health. 2026 Mar 24;14:1752273. doi: 10.3389/fpubh.2026.1752273 (PMC13133557; doi:10.3389/fpubh.2026.1752273)
Supplement: Supplementary file 1 [file Data_sheet_1.pdf]

We have now included the full "Health Literacy Assessment Scale for Unintentional Injuries in Young Children" as Supplementary File 1. Everyone can scan the QR code to view the scale.
