# Supplementary material for: Analysis of the current status and influencing factors of health literacy regarding unintentional injuries in young children among parents in ethnic regions of Western China
Source: Front Public Health. 2026 Mar 24;14:1752273. doi: 10.3389/fpubh.2026.1752273 (PMC13133557; doi:10.3389/fpubh.2026.1752273)
Supplement: Supplementary file 2 [file Data_sheet_2.pdf]

Table 1A. Univariate Analysis of Health Literacy by Child and Parent Demographic Characteristics (n=2984)

Variable	Total n (%)	Adequate HL n (%)	Inadequate HL n (%)	χ^2	P-value
**Preschool grade level**				10.948	0.027
Nursery class	199 (6.67)	98 (49.25)	101 (50.75)		
Junior class	653 (21.88)	345 (52.83)	308 (47.17)		
Middle class	1049 (35.15)	571 (54.43)	478 (45.57)		
Senior class	1004 (33.65)	589 (58.67)	415 (41.33)		
Preschool class	79 (2.65)	43 (54.43)	36 (45.57)		
**Parent's gender**				10.537	0.001
Male	584 (19.57)	291 (49.83)	293 (50.17)		
Female	2400 (80.43)	1357 (56.54)	1043 (43.46)		
**Parent's age (years)**				3.613	0.461
20–30	651 (21.82)	352 (54.07)	299 (45.93)		
31–40	2044 (68.50)	1125 (55.04)	919 (44.96)		
41–50	224 (7.51)	130 (58.04)	94 (41.96)		
51–60	52 (1.74)	26 (50.00)	26 (50.00)		
≥60	13 (0.44)	7 (53.85)	6 (46.15)		
**Parental education level**				51.043	<0.001
Junior high school and below	429 (14.38)	200 (46.62)	229 (53.38)		
High school / Secondary technical	638 (21.38)	304 (47.65)	334 (52.35)		
University / College	1788 (59.92)	1032 (57.72)	756 (42.28)		
Master's degree or above	129 (4.32)	97 (75.19)	32 (24.81)		
**Parental marital status**				1.631	0.442
Married	2890 (96.85)	1585 (54.84)	1305 (45.16)		
Divorced	83 (2.78)	43 (51.81)	40 (48.19)		
Widowed	11 (0.37)	5 (45.45)	6 (54.55)		
**Number of children**				9.484	0.009
1	1600 (53.62)	843 (52.69)	757 (47.31)		
2	1318 (44.17)	758 (57.51)	560 (42.49)		
≥3	66 (2.21)	34 (51.52)	32 (48.48)		
**Number of permanent residents**				2.893	0.235
2	123 (4.12)	70 (56.91)	53 (43.09)		
3–4	1979 (66.32)	1101 (55.63)	878 (44.37)		
≥5	882 (29.56)	473 (53.63)	409 (46.37)		

Table 1B. Univariate Analysis of Health Literacy by Family Socioeconomic and Training-Related Characteristics (n=2984)

Variable	Total n (%)	Adequate HL n (%)	Inadequate HL n (%)	χ^2	P-value
**Residential area**				32.217	<0.001
Urban	2374(79.56)	1356 (57.12)	1018 (42.88)		
Rural	610 (20.44)	279 (45.74)	331 (54.26)		

Variable	Total n (%)	Adequate HL n (%)	Inadequate HL n (%)	χ²	P-value
**Parent's occupation**				78.871	<0.001
Farmer	183 (6.13)	71 (38.80)	112 (61.20)		
Government official	106 (3.55)	70 (66.04)	36 (33.96)		
Corporate employee	713 (23.93)	440 (61.71)	273 (38.29)		
Public institution staff	441 (14.78)	235 (53.29)	206 (46.71)		
Self-employed / Business owner	426 (14.28)	254 (59.62)	172 (40.38)		
Retired	21 (0.70)	13 (61.90)	8 (38.10)		
Unemployed	213 (7.14)	95 (44.60)	118 (55.40)		
Freelance	575 (19.27)	310 (53.91)	265 (46.09)		
Other	306 (10.25)	152 (49.67)	154 (50.33)		
**Residential floor area (m²)**				29.228	<0.001
<61	80 (2.68)	36 (45.00)	44 (55.00)		
61–80	278 (9.32)	133 (47.84)	145 (52.16)		
81–100	977 (32.74)	508 (52.00)	469 (48.00)		
101–120	893 (29.93)	500 (55.99)	393 (44.01)		
>120	756 (25.34)	470 (62.17)	286 (37.83)		
**Monthly household income (Yuan)**				28.690	<0.001
<5000	617 (20.68)	300 (48.62)	317 (51.38)		
5000–10000	967 (32.41)	513 (53.05)	454 (46.95)		
10001–15000	641 (21.48)	362 (56.47)	279 (43.53)		
15001–20000	364 (12.20)	214 (58.79)	150 (41.21)		
>20000	395 (13.24)	248 (62.78)	147 (37.22)		
**Relevant knowledge training experience**				124.852	<0.001
Yes	894 (29.96)	614 (68.68)	280 (31.32)		
No	2090 (70.04)	1029 (49.23)	1061 (50.77)		
**Self-directed learning experience**				191.308	<0.001
Yes	1570 (52.61)	1028 (65.48)	542 (34.52)		
No	1414 (47.39)	616 (43.56)	798 (56.44)		
**Willingness to receive training**				6.614	0.010
Yes	2648 (88.74)	1470 (55.51)	1178 (44.49)		
No	336 (11.26)	165 (49.11)	171 (50.89)		
